# The Evaluation of *FGFR1*, *FGFR2* and *FOXO1* in Orofacial Cleft Tissue

**DOI:** 10.3390/children9040516

**Published:** 2022-04-06

**Authors:** Jana Goida, Mara Pilmane

**Affiliations:** Institute of Anatomy and Anthropology, Riga Stradins University, LV-1010 Riga, Latvia; mara.pilmane@rsu.lv

**Keywords:** cleft lip and palate, *FGFR1*, *FGFR2*, *FOXO1*

## Abstract

Although cleft lip with or without cleft palate (CL/P) is one of the most common congenital anomalies worldwide, the morphopathogenesis of non-syndromic orofacial clefts is still unclear. Many candidate genes have been proposed to play a causal role; however, only a few have been confirmed, leaving many still to be assessed. Taking into account the significance of *FGFR1*, *FGFR2* and *FOXO1* in embryogenesis, the aim of this work was to detect and compare the three candidate genes in cleft-affected lip and palatine tissue. Ten soft tissue samples were taken during cheiloplasty and veloplasty. The signals of the candidate genes were visualized using chromogenic in situ hybridization and analyzed using a semi-quantitative method. No statistically important difference in the distribution of *FGFR1*, *FGFR2* and *FOXO1* between neither the patients’ lip and vomer mucosa nor the control group was observed. Statistically significant very strong and strong correlations were found between genes in the lip and palatine tissue. The expression of *FGFR1*, *FGFR2* and *FOXO1* in cleft-affected lip and palatine tissue seems to be highly individual. Numerous intercorrelations between the genes do not exclude their role in the possible complex morphopathogenesis of orofacial clefts.

## 1. Introduction

In human development, facial primordia, consisting of frontonasal prominences, maxillary and mandibular prominences, develops by the end of the 4th week due to migrating cranial neural crest cells [1]. Due to local thickening in the frontonasal prominences, nasal placodes form, which, during the 5th week invaginate, form nasal pits. By doing so, that gives rise to lateral and medial nasal prominences. In the following two weeks, maxillary prominences increase in size and grow medially, thus compressing the medial nasal prominences towards the midline. Afterwards, both fuse and the upper lip is formed. Due to the medial growth of maxillary prominences, the medial nasal prominences merge, forming the intermaxillary segment [2]. Development of the secondary palate begins during the 6th week with the appearance of palatine shelves from the maxillary prominence, which are directed obliquely downward along the sides of the tongue. Due to the growth of the mandibula, the tongue moves downwards, allowing the palatine shelves to ascend, thus attaining a horizontal position above the dorsum of the tongue [3]. During the 8th week, the palatine shelves grow towards each other until the medial edge epithelia (MEE) fuse at the mid-line, thus forming the midline edge seam (MES), which disappears afterwards. The process of palatine fusion is completed by the 12th week of gestation [1,3]. The development of orofacial structures is an intricate multistep process during which interaction between mesodermal- and neural crest-derived mesenchyme and ecto- and endodermal-derived epithelium plays a vital role [4]. Any disruption in the cell migration, proliferation, differentiation, fusion and apoptosis during orofacial development could lead to anomalies, such as orofacial clefts [1].

Cleft lip with or without cleft palate (CL/P) is one of the most common congenital anomalies worldwide. Depending on the region and ethnicity, the incidence varies from approximately 1 in 500 to 2500 births [1,5]. Furthermore, CL/P has been observed more frequently among males than females [6]. Socioeconomic status is another factor capable of affecting the incidence [7,8]. The majority of CL/P are thought to be non-syndromic (approximately 70%), whilst only about 30% syndromic [1]. Due to many studies, it is clear that orofacial clefts are a multifactorial pathology. Some of the environmental risk factors are maternal smoking, teratogenic medication (for instance, anticonvulsants), folate deficiency and, in some studies, alcohol [9,10,11,12]. Even though throughout the years many candidate genes have been proposed to have a causal role in the morphopathogenesis of non-syndromic orofacial clefts [13,14,15], unfortunately, only a few, for example *IRF6* [16,17], have been confirmed. Other potential candidate genes, such as genes from the FGF and FOX families, remain to be assessed.

The Fibroblast Growth Factor family (FGF) consists of 18 secreted proteins, 4 intracellular non-signaling proteins, 4 tyrosine kinase FGF receptors (FGFR) and a non-tyrosine kinase receptor (FGFRL1). Ligand binding specificity of FGFRs is regulated by immunoglobulin-like domains II and III and the linker region between these domains. Immunoglobulin-like domain I and acidic box are believed to inhibit ligand binding [18]. *FGFR1*-*FGFR3* have two additional splice variants of immunoglobulin-like domain III, called IIIb and IIIc. Epitheliocytes preferentially express the IIIb form; however, they are often activated by ligands from adjacent mesenchyme. On the other hand, mesenchymal cells express IIIc isoform but bind ligands usually expressed in the near epithelium. This configuration functions as a safeguard to avoid accidental autocrine stimulation [19]. FGFs are one of the factors involved in the regulation of cell proliferation, differentiation, migration and apoptosis. Therefore, members of the FGF family are not only vital for embryogenesis but also organogenesis, angiogenesis, wound healing and tumorigenesis [20]. Multiple studies have detected *FGFR1* and *FGFR2* expression in orofacial cleft tissue, therefore indicating a possible role in orofacial cleft morphopathogenesis [21,22,23,24]. Using peripheral blood, the role of *FGFR1* and *FGFR2* gene polymorphisms in orofacial cleft development has been researched, furthering the knowledge of orofacial cleft formation [23,25,26,27]. Moreover, mutations in *FGFR1* and *FGFR2* genes have been established in the pathogenesis of some syndromes in which clefts are present, such as Apert syndrome (*FGFR2*), Crouzon syndrome (*FGFR2*, *FGFR3*), Hartsfield syndrome (*FGFR1*) and Kallmann syndrome (*FGFR1*, FGF8) [18].

The FOXO subfamily belongs to the Forkhead Box (FOX) gene family, which consists of 50 members and two pseudogenes (*FOXO1B*, *FOXO3B)* [28]. Overall, the FOXO subfamily has an important role in regulating genes involved in DNA damage repair, cell metabolism and oxidative stress resistance, as well as in cell cycle arrest and apoptosis [29]. During embryogenesis, high expression of *FOXO1* has been detected in developing embryonic vasculature, indicating that *FOXO1* plays a significant role in embryonic angiogenesis [30,31]. Furthermore, *FOXO1* has been recognized as an essential transcriptional factor for enamel biomineralization [32]. Moreover, *FOXO1* has been observed in lip and palatine tissue, hence its potential role in lip and palatine development cannot be excluded [24,33]. Whilst *FOXO1* polymorphism has been examined in other diseases, for instance, type 2 diabetes [34], no studies were found concerning orofacial clefts.

Even though the dysregulated function of *FGFR1*, *FGFR2* and *FOXO1* has been examined in non-syndromic orofacial clefts, there has been limited research regarding the comparison of these factors in one individual’s two separate locations—cleft-affected lip tissue and cleft-affected palatine tissue. Therefore, taking into account the significance of *FGFR1*, *FGFR2* and *FOXO1* in embryogenesis, as well as its potential influence on the complex mesenchymal and epithelial interplay, the aim of this study was to examine the appearance and distribution of *FGFR1*, *FGFR2* and *FOXO1*, as well as deduce any possible correlations between the three genes in cleft-affected lip and palatine tissue.

## 2. Materials and Methods

### 2.1. Information about the Patients

This study was conducted in accordance with the 1964 Declaration of Helsinki, and the study protocol was approved by the Ethical Committee of Riga Stradins University on 17 January 2013 and 24 September 2020. (Nr.6-1/10/11). After a detailed explanation of the study, written informed consent was obtained from all the patients’ parents.

The study group consisted of 10 children (8 males and 2 females) diagnosed with cheilognathouranoschisis dextra, sinistra or bilateralis. The inclusion criteria were as follows: diagnosed with non-syndromic unilateral or bilateral cleft lip, alveolar ridge and palate, at the time of first surgery aged before primary dentition, without periodontal diseases or any other pathology impeding the patient from receiving cheiloplasty and veloplasty. The 3rd and 10th child were included as borderline cases (Table 1). The samples of lip mucosa were collected during cheiloplasty, but vomer mucosa during veloplasty by the same surgeon at the Institute of Stomatology, Riga Stradins University, Latvia. In 5 out of 10 cases, no additional remarks were made by the physician. Orofacial clefts in family history were noted in 2 cases; however, one child had other anomalies besides CL/P. In detail, all the information about the patients is summarized in Table 1.

The control group consisted of 4 males and 2 females, aged 7–19 years. Three lip mucosa samples were obtained during superior labial frenectomy due to hypertrophic frenula labii superioris. The inclusion criteria were: diagnosed with hypertrophic upper lip frenulum, no orofacial cleft in anamnesis or family history, without inflammation or any other pathology present. In addition, three soft palatine samples were obtained from cadavers aged 17–19 years at the Institute of Anatomy and Anthropology. The cause of death had no association with any anomalies, inflammation or other pathology.

### 2.2. Chromogenic In Situ Hybridization

Even though the correlation between chromogenic in situ hybridization (CISH), fluorescence in situ hybridization (FISH) and real-time polymerase chain reaction (real-time PCR) are high, taking into account the many advantages of CISH, such as simultaneous observation of tissue morphology and CISH signals without the need of an expensive fluorescence microscope, it was decided to use CISH to visualize the potential mRNA copies of the candidate genes [35,36,37,38].

Immediately after cheiloplasty or veloplasty, tissue samples were collected and fixed for a day in a mixture of 2% formaldehyde and 0.2% picric acid in 0.1M phosphate buffer (pH 7.2). Afterwards, the samples were rinsed in Tyrode’s buffer (content: NaCl, KCl, CaCl_2_·2H_2_O, MgCl_2_·6H_2_O, NaHCO_3_, NaH_2_PO_4_·H_2_O, glucose), containing 10% saccharose, for 12h and then embedded into paraffin. Tissue samples were registered and given randomized codes. Furthermore, except for patients’ history (Table 1), no other information about the patients was available to the researchers and laboratory assistants.

In the study, ZytoDot 2C CISH Implementation Kit (ZytoVision GmbH, Bremerhaven, Germany) with FGFR1, FGFR2 and FOXO1 probes were used. Pretreatment was done using standard laboratory methods. Denaturation and hybridization began with applying 10 microliters of the probe onto each pretreated specimen using a pipette. Afterwards, specimens were covered with an 18 × 18 mm coverslip and placed on a hot plate (79 °C) for 5min. Then, they were transferred to a humidity chamber and hybridized overnight at 37 °C, ensuring that specimens did not dry out. The next day the slides were submerged first in SSC wash buffer then TBS wash buffer to remove the coverslips. Further, the specimens underwent the next steps of the CISH procedure as per the guidelines of the manufacturer. Slides were transferred into a staining jar, washed under cold running water for 2 min, dehydrated with 100% ethanol and then incubated in xylene. Avoiding trapped air bubbles, the coverslips were re-attached and the specimens were analyzed under a light microscope. Turquoise-colored dots indicated the targeted gene region, and bright red indicated control. Hybridized probe fragments were visualized after counterstaining the nucleus with a nuclear dye. In the interphase of normal cells or cells without aberrations, two distinct dots were expected to appear in the cell’s nuclei.

The specimens were analyzed using a semi-quantitative scoring system [39] by two independent morphologists who were blinded to the sample identification. The results were assessed by grading the appearance of turquoise-colored dots in at least five randomly selected fields of view at 1000× magnification using immersion oil. The signals from the turquoise probes were evaluated in the epithelium, connective tissue and endothelium of lip and vomer mucosa. Structures were labelled as follows: 0, no turquoise signals (copies) detected (0%); 0/+, occasional turquoise signals detected (12.5%); +, few copies detected (25%); +/++, few to moderate copies detected (37.5%); ++, moderate number of turquoise signals detected (50%); ++/+++, moderate to numerous copies detected (62.5%); +++, numerous turquoise signals detected in the visual field (75%) [40].

For visual illustration, Leica LEITZ DM RB microscope, Euromex Scientific Camera DC.20000i, and the image processing and analysis software ImageFocusAlpha (Euromex Microscopen bv, Arnhem, The Netherlands) were used.

### 2.3. Statistical Analysis

IBM SPSS Statistics Version 27 (IBM Company, Armonk, New York, NY, USA) was used for data analyses. The results from semi-quantitative evaluation were transformed into numerical form, for example, 0 equals to 0, 0/+ equals to 0.5, + equals to 1, et cetera. Statistical significance was calculated with the Mann–Whitney U test. For correlations analyses, the Spearman’s rank correlation coefficient was used, and the R-value was interpreted as follows: R = 0.00–0.19, a very weak correlation; R = 0.20–0.39, a weak correlation; R = 0.40–0.59, a moderate correlation; R = 0.60–0.79, a strong correlation; R = 0.80–1.0, a very strong correlation. For both tests, the *p*-value of <0.05 was considered statistically significant.

## 3. Results

The presence of genes, as turquoise-colored dots, was observed in the majority of cases, with the exception of four lip tissue samples and two palatine (Table 2). However, the first and seventh patients had no gene signals in either lip or vomer mucosa. Overall, gene-mRNA-copy-containing cells were observed more in the epithelium, followed by the connective tissue and then endothelium.

**Figure 1:**
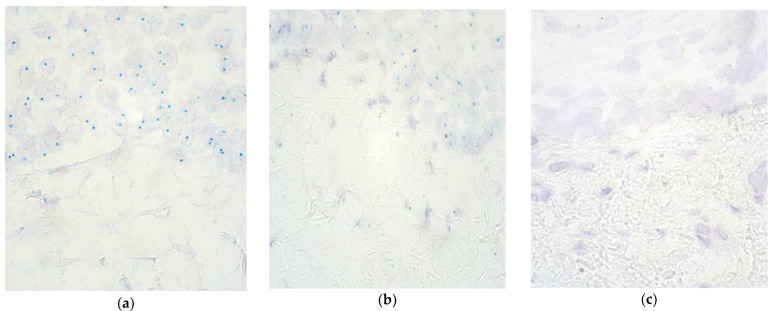
Chromogenic in situ hybridization micrographs of *FGFR1* in cleft-affected lip and palatine tissue and control subject at 1000× magnification, using immersion oil. (**a**) Numerous (+++) gene signals in lip epithelium and no gene copies in lip connective tissue in a 3.5-month-old patient. (**b**) Numerous (+++) gene signals in palatine epithelium and a few (+) gene copies in palatine connective tissue in a 10-month-old patient. (**c**) No (0) gene signals in the control group epithelium and connective tissue of a 10-year-old child.

*FGFR1* presence in the epithelium of vomer mucosa varied greatly from no copies detected (0) to numerous (+++); however, in the lip epithelium, the majority of the cases (8 out of 10) had no turquoise dotted cells (0), and only two tissue samples contained numerous gene copies (+++) (Table 2, Figure 1a,b). In fact, *FGFR1* presence in the lip mucosa was observed only in two patients’ tissue samples. The number of gene-containing cells was similar between the connective tissue and endothelium of the lip and vomer mucosa. In terms of controls, across all locations, no gene-containing cells (0) were observed, with the exception of one tissue sample’s epithelium, in which a few (+) gene signals were detected (Figure 1c). Due to similar results, the control data of lip and palatine mucosa samples were summarized in the form of a mean value (Table 2).

**Figure 2:**
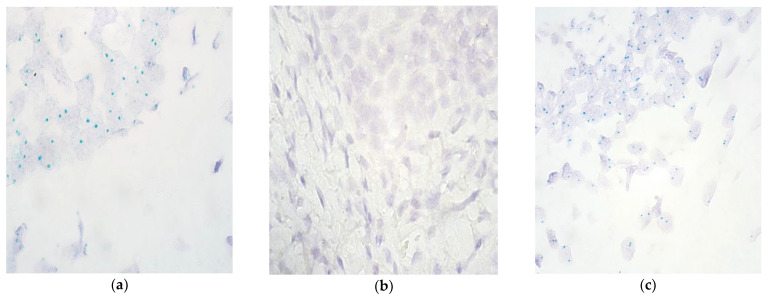
Chromogenic in situ hybridization micrographs of *FGFR2* in cleft-affected lip and palatine tissue and control subject at 1000× magnification, using immersion oil. (**a**) Numerous (+++) gene signals in lip epithelium and moderate amount (++) of gene copies in lip connective tissue in a 3-month-old patient. (**b**) No (0) gene signals in palatine epithelium and connective tissue in a 15-month-old patient. (**c**) Numerous (+++) gene signals in the control group epithelium of a 7-year-old child.

The majority of epitheliocytes in the lip and vomer mucosa (6 out of 10 and 7 out of 10, respectively) contained the *FGFR2* gene, ranging from occasional copies (0/+) to numerous (+++) (Table 2, Figure 2a,b). In the connective tissue and endothelium of lip and vomer mucosa, the range of observed *FGFR2* signals was the same (from 0 to +/++). However, more lip tissue samples had 0 gene signals in connective tissue (7 out of 10) and endothelium (8 out of 10) compared to palatine (5 out of 10 and 7 out of 10, respectively). In the control group, the range of observed *FGFR2* signals in the epithelium was the same as in the study group; however, it was narrower in the connective tissue and endothelium with the highest value of 0/+ and +, respectively (Figure 2c).

**Figure 3:**
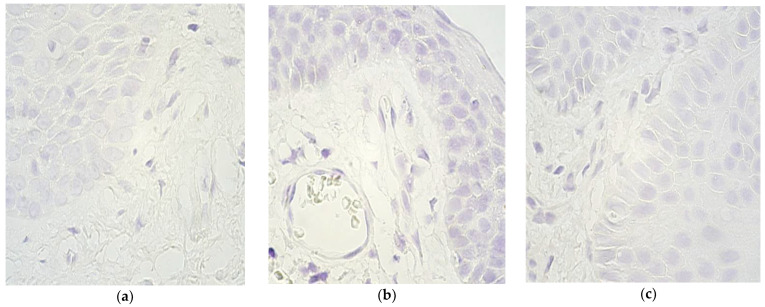
Chromogenic in situ hybridization micrographs of *FOXO1* in cleft-affected lip and palatine tissue and control subject at 1000× magnification, using immersion oil. (**a**) No (0) gene signals in lip epithelium and connective tissue in a 3-month-old patient. (**b**) No (0) gene signals in palatine epithelium, connective tissue and endothelium in a 10-month-old patient. (**c**) No (0) gene signals in the control group epithelium and connective tissue of a 7-year-old child.

Across all locations, more lip tissue samples contained *FOXO1* than palatine, and the range was slightly wider for lip mucosa compared to vomer (Table 2, Figure 3a,b). Furthermore, the majority of palatine connective tissue cases (9 out of 10) had no *FOXO1* expressing cells (0), whilst only one tissue sample had occasional *FOXO1* signals (0/+). In the control group, across all locations, no *FOXO1* copies were detected (Figure c).

No statistically significant difference in the distribution of a particular gene between neither the patients’ lip mucosa and vomer mucosa nor the control group and lip or palatine mucosa was observed (Table 3). Furthermore, the *p*-value varied greatly from 0.280 to 1.000.

In the lip mucosa, a positive very strong and strong correlation, which was statistically significant, was observed in 7 and 10 out of 36 pairs, respectively (Supplementary file, Table S1). In the vomer mucosa, five statistically important pairs with very strong correlations and four pairs with strong correlations were observed (Supplementary file, Table S2). Overall, in both locations, the R-value varied greatly, with six pairs having a statistically insignificant negative weak correlation in the lip, but 12 negative correlations in the palatine tissue samples (Supplementary file, Tables S1 and S2).

Overall, in lip and palatine tissue samples, a very strong correlation between *FGFR1* in the connective tissue and epithelium (R = 0.994 and R = 0.837, respectively) and endothelium (R = 1.000 and R = 0.855, respectively) was observed (Table 4). In both lip and palatine tissue, a strong association was found between *FGFR1* in endothelium and *FOXO1* in epithelium (R = 0.651 and R = 0.679, respectively); however, not only was a very strong correlation between *FGFR1* in endothelium and *FGFR1* in lip’s epithelium (R = 0.994) found, but also a strong correlation in palatine epithelium (R = 0.725). Further, in both locations, *FOXO1* in endothelium was very strongly associated with *FOXO1* in epithelium (R = 0.915 and R = 0.861) as well as with *FOXO1* in lip’s connective tissue (R = 0.995) but strongly associated with *FOXO1* in palatine connective tissue (R = 0.667). Lastly, a very strong correlation was observed between *FGFR2* in connective tissue and endothelium of both lip and vomer mucosa (R = 0.855 and R = 0.802, respectively), but a strong correlation in lip’s epithelium (R = 0.652) and a very strong correlation in the epithelium of palate (R = 0.811).

## 4. Discussion

Morphopalatogenesis is a complex cascade of events during which precise spatiotemporal expression of different genes is required [41]. Furthermore, a key factor is the epithelial–mesenchymal interaction [4].

Many studies have shown that normal palatogenesis requires a fine balance of protein and gene expression. For instance, Pfeiffer syndrome is caused by a gain-of-function missense mutation in *FGFR1* or activating mutation in *FGFR2* [18,42]. Similarly, due to the gain-of-function mutation in *FGFR2,* Crouzon syndrome arises [43]. Additionally, the development of orofacial clefts is associated with *FGFR1* loss-of-function mutations and haploinsufficiency [44,45,46]. Lastly, a study showed that *FOXO1* knockdown in the palatal shelf organ model resulted in inhibited medial edge epithelium (MEE) cell apoptosis [33]. Interestingly, a case study of two children with Apert syndrome proved that identical mutations do not always result in identical clinical manifestation [47]. Even though in our study group the expression of *FGFR1*, *FGFR2* and *FOXO1* in the lip and palatine tissue varied greatly, no statistically significant difference was observed between the two locations or the control. Whilst 2 out of 10 patients had no gene expression in lip or vomer mucosa, two other patients had low expression of *FGFR1* and *FGFR2* in palatine tissue, but no gene expression in the lip. Overall, this could indicate different causative individual mechanisms for cleft development between the patients in our study group.

As mentioned before, mesenchymal tissue expresses IIIc splice variants of *FGFR1* and *FGFR2*; however, not the epithelium- IIIb variants [18]. Interestingly, the results of a study conducted by Yu et al. revealed that during normal palatogenesis of mice, *FGFR1* was expressed throughout the entire palatine mesenchyme, but not in the epithelium. However, depending on the stage of development, *FGFR2* was primarily observed in the epithelium [21]. Overall, in our study, the epithelium of lip and vomer mucosa contained the most *FGFR1*-expressing cells, followed by the connective tissue and lastly the endothelium. This pattern of expression is in line with another study’s results, in which *FGFR1*, as well as *FGFR2* and *FOXO1*, were observed in only cleft-affected lip tissue [24]. A study carried out by Wang et al. showed that deletion of *FGFR1* in mice neural crest cells resulted in delayed cell proliferation in the epithelium as well as mesenchyme, failure in palate shelf elevation and compromised deterioration of the MEE [48]. Similarly, another research work revealed that mesenchyme-specific disruption of both *FGFR1* and *FGFR2* led to cleft palate development due to disrupted palatal shelf elevation [21]. Conversely, deletion of *SPRY2*, an inhibitor of FGF signaling, intensified FGF signaling, resulting in increased cellular proliferation in the palate shelves as well as cleft palate [49]. This is further supported by another study in which increased Ki-67 in the cleft-affected lip tissue samples was observed [50]. Furthermore, *FGFR1* signaling has been shown to be a key inhibitor of endothelial-to-mesenchyme transition (EndTM), and thus a critical anti-fibrotic agent. In addition, it plays a vital role in normal vascular homeostasis [51,52]. Moreover, through the activation of NF-κB, *FGFR1* is involved in the regulation of inflammatory responses. Studies have shown that by inhibiting *FGFR1*, the inflammation in tissue decreases [53,54,55]. For instance, the blocking of *FGFR1* in hepatic stellate cells resulted in decreased pro-inflammatory cytokine release, cell proliferation and fibrosis [53]. Similarly, cell-specific deletion of *FGFR1* in the cerebellum of an experimental autoimmune encephalomyelitis model revealed reduced expression of inflammatory cytokines, less axonal damage and myelin loss and lowered inflammation [54]. Overall, our results suggest increased expression of *FGFR1* in the epithelium and decreased expression in the endothelium, thus indicating a possible role for increased cell proliferation, fibrosis and inflammation in cleft-affected tissue. Interestingly, a study concerning M1 and M2 macrophages and TNFα and another research about different cytokines in cleft-affected lip tissue samples reported increased tissue inflammation together with persistent elevation of the protective mechanisms in cleft-affected tissue [50,56].

Interaction between FGF10 and *FGFR2b* is important for normal palatogenesis [57]. Interaction between FGFs and Foxi3 in ectodermal organogenesis has also been addressed [58,59,60]. Whilst FGF10 is expressed in palatine mesenchyme, *FGFR2b* is mainly expressed in palatine epithelium, and *FGFR2c* in mesenchyme [61]. *FGFR2b* and FGF10 null mice revealed significantly reduced cell proliferation in both epithelium and mesenchyme. Furthermore, results from Rice et al. suggested that the epithelium supports mesenchyme proliferation. Therefore, this provides evidence that signals are sent not only from the mesenchyme to the overlaying epithelium, but also vice versa [57]. In contrast, continued expression of *FGFR2* seems to result in increased cell proliferation in palatal shelf mesenchyme and delayed elevation of palatine shelves [43,62]. In our research, the epithelium of both lip and palatine tissue contained the most *FGFR2*-expressing cells. This is in line with the aforementioned mice study, in which during normal palatogenesis *FGFR2* primarily was expressed in the epithelium, as well as another study, in which *FGFR2* was examined in cleft-affected lip tissue [21,24]. However, in our study group, there were cases in which no *FGFR2* expression in either epithelium or mesenchyme was observed. Overall, our results indicate a disruption in epithelial-mesenchyme interaction in the cleft-affected tissue, as well as the possibility that the mechanisms of cleft development differ between our patients.

Examining *FOXO1* expression in mice from 7.0 dpc to 18.5 dpc revealed that the expression is highly dynamic throughout the whole embryo. For example, depending on the stage of development, the expression was observed in neural crest cells of the first two branchial arches, epithelium of the medial nasal process and lip [63]. In our study, more lip tissue samples showed *FOXO1* expression than palatine; although, in both lip and vomer mucosa, the expression was strongest in the epithelium. However, identically to *FGFR1* and *FGFR2* expression, no statistically significant difference in the distribution between lip, palatine and control tissue samples was observed. Research carried out by Xu et al. revealed that *BAG6* promotes p300’s mediated *FOXO1* acetylation in MEE cells, which in turn enhances FasL/caspase-3 activity and MEE apoptosis, thus ensuring proper palate fusion [33]. Interestingly, in Apert syndrome, an upregulation of PI3L/Akt can be observed which results in decreased *FOXO1* [64]. A hard and soft palate has also been observed in cleidocranial dysplasia, which is a rare congenital skeletal dysplasia caused by haploinsufficiency in the *RUNX2* gene [65]. A study carried out by Teixeira et al. showed that *FOXO1* drives mesenchymal cells towards osteogenic differentiation, and one of the mechanisms through which that is achieved is by directly interacting with the promoter of *RUNX2*, which is a key osteogenic transcription factor, and regulating its expression. Silencing *FOXO1* resulted in the decreased expression of *RUNX2* and impaired craniofacial development [66]. Another research work conducted by Shirai et al. showed that *RUNX2* expression is necessary during the intramembranous ossification process of neural crest-derived cells. In this study, *RUNX2*$\begin{array}{c} +/-\\ wnt1 \end{array}$ mouse embryos lacked the secondary palate [67]. A tumorigenic role of *RUNX2* has been demonstrated as well [68,69]. In addition, in skeletal development and the pathogenesis of craniosynostosis, the reciprocal regulation of *RUNX2* and FGF signaling plays an important role [70,71,72]. Moreover, *FOXO1* is an essential regulator of endothelial proliferation and metabolism. *FOXO1* deletion in mice resulted in increased endotheliocyte proliferation which interfered with coordinated sprouting thus causing hyperplasia and blood vessel enlargement. On the other hand, overexpression of *FOXO1* led to restricted vascular expansion, thereby causing blood vessel thinning and hypobranching. Furthermore, by reducing glycolysis and mitochondrial respiration, *FOXO1* diminishes metabolic activity, thus providing potential protection against oxidative stress [73]. This study’s results suggest an overall decreased *FOXO1* expression in cleft-affected lip and palatine tissue, therefore a greater risk for oxidative stress and cell damage, which consequently leads to inflammation. Impaired skeletal development could have interfered with the maxilla and hard palate formation, thereby disrupting primary and secondary palate formation. In addition, data reveals that keratinocyte-specific deletion of *FOXO1* leads to impaired re-epithelialization and reduced fibroblast and mesenchymal stem cell proliferation during wound healing, resulting in impaired wound healing [74,75].

Taking into account the aforementioned information and the importance of epithelial/mesenchymal expression of different genes, particularly alternative splice variants of FGFRs and reciprocal expression of interacting FGFs in development [18], we expected statistically significant correlations between *FGFR1*, *FGFR2* and *FOXO1*. However, our results only revealed strong and very strong positive statistically important correlations between one gene type and different tissue layers, for instance between *FGFR1* in epithelium and *FGFR1* in connective tissue and endothelium. However, there was one exception—a strong positive correlation was observed between *FGFR1* and *FOXO1* in all lip tissue layers. Our results are partly in agreement with another study, in which a strong correlation between *FGFR1* in the epithelium and connective tissue and *FGFR2* in the epithelium and connective tissue was observed [24]. In our opinion, the positive correlations within the boundaries of one gene but different tissue layers could suggest that the disruption of gene function is not limited to one tissue layer and/or normal gene expression in the nearby layers is changed as a means of protection. On the other hand, the positive correlation between *FGFR1* and *FOXO1* could be explained by their role in vascular homeostasis and *FOXO1* regulation of metabolism mentioned before.

Our study had some limitations. Whilst *FGFR1*, *FGFR2* and *FOXO1* have been examined in cleft-affected lip tissue, to our knowledge, this was the first study in which these three genes were evaluated between the cleft-affected lip and palatine tissue. Therefore, for instance, a comparison of results is not possible. Moreover, both the study group and control group are too small. This could have affected the statistical significance of our results and may have interfered with the general applicability of our findings. The oral development could be divided into two major periods—before and during milk dentition, and mixed and permanent dentition. It has been suggested that the distribution of genes, growth factors and cell apoptosis could be influenced by age in orofacial clefts [76]. Furthermore, other studies have reported an intricate mix of environmental and genetically driven changes in gene expression with age [77,78]. Hence, the major age difference between the study and control group in our research is a significant limitation. Furthermore, the *FGFR2* CISH probe used in this study could not distinguish between *FGFR2b* and *FGFR2c* forms.

Moving further, for future studies, we would recommend larger study and control groups. Additionally, combining different methods to evaluate *FGFR2* isoforms and FGFs could greatly impact the understanding of the roles of *FGFR1*, *FGFR2* and *FOXO1* in orofacial cleft development.

## 5. Conclusions

The expression of *FGFR1*, *FGFR2* and *FOXO1* in cleft-affected lip and palatine tissue seems to be highly individual, suggesting a possible difference in postnatal cleft morphopathogenetic mechanisms.

Numerous intercorrelations between *FGFR1*, *FGFR2* and *FOXO1* do not exclude their role in the possible complex morphopathogenesis in each individual affected by orofacial clefts.

## Figures and Tables

**Table 1:** Information about the patients.

No.	Gender	Diagnosis	Procedure	Age (Months)	Remarks
1.	M	Cheilognathouranoschisis dextra	Right cheiloplasty	3	-
			Veloplasty	10	
2.	M	Cheilognathouranoschisis dextra	Right cheiloplasty	3	-
			Veloplasty	9	
3.	M	Cheilognathouranoschisis sinistra	Left cheiloplasty	3	Mother with a cleft lip and palate.
			Veloplasty	8	
4.	M	Cheilognathouranoschisis sinistra	Left cheiloplasty	3.5	Paracetamol had been used during pregnancy. Epilepsy in family tree. Father a regular smoker.
			Veloplasty	15	
5.	F	Cheilognathouranoschisis sinistra	Left cheiloplasty	4	-
			Veloplasty	9	
6.	F	Cheilognathouranoschisis dextra	Right cheiloplasty	4	-
			Veloplasty	8	
7.	M	Cheilognathouranoschisis dextra	Right cheiloplasty	4	Hepatitis B during the pregnancy. Cleft lip and palate in the family tree.
			Veloplasty	10	
8.	M	Cheilognathouranoschisis sinistra	Left cheiloplasty	4.5	Down syndrome present in family history.
			Veloplasty	10	
9.	M	Cheilognathouranoschisis sinistra	Left cheiloplasty	5	-
			Veloplasty	10	
10.	M	Cheilognathouranoschisis bilateralis	Bilateral cheiloplasty	13	Multiple anomalies including congenital heart failure.
			Veloplasty	36	

Abbreviations: No.—patient’s number, M—male, F—female.

**Table 2:** The relative number of *FGFR1*, *FGFR2* and *FOXO1* gene signals in the cleft-affected lip and palatine tissue samples as well as in the control group.

**Lip Mucosa**									
**Patient’s No.**	**Epithelium**			**Connective Tissue**			**Endothelium**		
	***FGFR1***	***FGFR2***	***FOXO1***	***FGFR1***	***FGFR2***	***FOXO1***	***FGFR1***	***FGFR2***	***FOXO1***
1.	0	0	0	0	0	0	0	0	0
2.	0	+++	0	0	+/++	0	0	+	0
3.	0	+++	0	0	0	0	0	0	0
4.	+++	0/+	+++	++	0	+	+	0	++
5.	0	++	+++	0	+/++	++/+++	0	0/+	++
6.	0	0	0	0	0	0	0	0	0
7.	0	0	0	0	0	0	0	0	0
8.	+++	0/+	++/+++	+	0	0/+	0/+	0	+
9.	0	+++	0/+	0	+	0	0	0	0
10.	0	0	0	0	0	0	0	0	0
**Mode**	0	0	0	0	0	0	0	0	0
**Control**	0	0	0	0	0/+	0	0	0	0
**Vomer Mucosa**									
**Patient’s No.**	**Epithelium**			**Connective Tissue**			**Endothelium**		
	***FGFR1***	***FGFR2***	***FOXO1***	***FGFR1***	***FGFR2***	***FOXO1***	***FGFR1***	***FGFR2***	***FOXO1***
1.	0	0	0	0	0	0	0	0	0
2.	+/++	+++	0	0	+/++	0	0	+	0
3.	0	+++	0	0	0/+	0	0	0/+	0
4.	++/+++	0	++/+++	+/++	0	0	+	0	0/+
5.	0	0/+	++/+++	0	0/+	0/+	0	0	0/+
6.	++	+	0	0/+	0	0	0	0	0
7.	0	0	0	0	0	0	0	0	0
8.	+++	+++	0/+	+/++	+	0	+/++	0/+	0
9.	0/+	+++	0	0	0/+	0	0	0	0
10.	0/+	0/+	0	0	0	0	0	0	0
**Mode**	0	+++	0	0	0	0	0	0	0
**Control**	0	0	0	0	0/+	0	0	0	0

Abbreviations: No.—patient’s number, *FGFR1*—fibroblast growth factor receptor 1, *FGFR2*—fibro-blast growth factor receptor 2, *FOXO1*—forkhead box O1.

**Table 3:** The statistical importance of the distribution of *FGFR1*, *FGFR2* and *FOXO1* genes between the patients’ lip and palatine mucosa as well as the control group and study group.

**Lip Mucosa vs. Vomer Mucosa**									
	**Epithelium**			**Connective Tissue**			**Endothelium**		
	***FGFR1***	***FGFR2***	***FOXO1***	***FGFR1***	***FGFR2***	***FOXO1***	***FGFR1***	***FGFR2***	***FOXO1***
Mann–Whitney U	65	56	42.5	54	55	39	51.5	54.5	42
*p*-Value	0.280	0.684	0.579	0.796	0.739	0.436	0.912	0.739	0.579
**Lip Mucosa vs. Control**									
	**Epithelium**			**Connective Tissue**			**Endothelium**		
	***FGFR1***	***FGFR2***	***FOXO1***	***FGFR1***	***FGFR2***	***FOXO1***	***FGFR1***	***FGFR2***	***FOXO1***
Mann–Whitney U	16	31	9	12	35	10.5	12	39.5	10.5
*p*-Value	1.000	1.000	0.371	0.692	0.635	0.469	0.692	0.313	0.469
**Vomer Mucosa vs. Control**									
	**Epithelium**			**Connective Tissue**			**Endothelium**		
	***FGFR1***	***FGFR2***	***FOXO1***	***FGFR1***	***FGFR2***	***FOXO1***	***FGFR1***	***FGFR2***	***FOXO1***
Mann–Whitney U	10	28.5	10.5	10.5	31	13.5	12	37.5	12
*p*-Value	0.469	0.875	0.469	0.469	1.000	0.811	0.692	0.428	0.692

Abbreviations: vs.—versus, *FGFR1*—fibroblast growth factor receptor 1, *FGFR2*—fibroblast growth factor receptor 2, *FOXO1*—forkhead box O1.

**Table 4:** Statistically significant correlations between factors in patients’ lip and palatine tissue samples based on Spearman’s correlation analyses.

**Factor 1**	**Factor 2**	**R_1_**	***p*-Value_1_**	**R_2_**	***p*-Value_2_**
**Very Strong Correlation**					
*FGFR1* in epithelium	*FGFR1* in connective tissue	0.994	0.000	0.837	0.002
*FGFR1* in connective tissue	*FGFR1* in endothelium	1.000	0.000	0.855	0.002
*FGFR2* in connective tissue	*FGFR2* in endothelium	0.855	0.002	0.802	0.005
*FOXO1* in epithelium	*FOXO1* in endothelium	0.915	0.000	0.861	0.001
**Very Strong-Strong Correlation**					
*FGFR1* in epithelium	*FGFR1* in endothelium	0.994	0.000	0.725	0.018
*FOXO1* in connective tissue	*FOXO1* in endothelium	0.995	0.000	0.667	0.035
*FGFR2* in epithelium	*FGFR2* in connective tissue	0.652	0.041	0.811	0.004
**Strong Correlation**					
*FOXO1* in epithelium	*FGFR1* in endothelium	0.651	0.042	0.679	0.031

Abbreviations: *FGFR1*—fibroblast growth factor receptor 1, *FGFR2*—fibroblast growth factor receptor 2, *FOXO1*—forkhead box O1. Note: R_1_ and *p*-Value_1_ indicate the calculated correlations between Factor1 and Factor2 in lip tissue samples; however, R_2_ and *p*-Value_2_ indicate those in palatine tissue samples.

## Data Availability

The data used and/or analyzed in this study are presented in the results section of the present re-search and attached Supplementary file.

## Supplementary Materials

The following supporting information is available online at: https://www.mdpi.com/article/10.3390/children9040516/s1, Table S1: Correlations between factors in patients’ lip tissue samples based on Spearman’s correlations analyses.; Table S2: Correlations between factors in patients’ palatine tissue samples based on Spearman’s correlations analyses.
