# Supplementary material for: The Evaluation of FGFR1, FGFR2 and FOXO1 in Orofacial Cleft Tissue
Source: Children (Basel). 2022 Apr 6;9(4):516. doi: 10.3390/children9040516 (PMC9032315; doi:10.3390/children9040516)
Supplement: Supplementary file 1 [file children-09-00516-s001.zip › children-1634484-supplementary.pdf]

Supplementary file

Table S1. Correlations between factors in patients' lip tissue samples based on Spearman's correlations analyses.

Factor 1	Factor 2	R	*p*-Value
**Very strong correlation**			
FGFR1-e	FGFR1-c	0.994	0.000
FGFR1-e	FGFR1-end	0.994	0.000
FOXO1-e	FOXO1-c	0.911	0.000
FOXO1-e	FOXO1-end	0.915	0.000
FGFR1-c	FGFR1-end	1.000	0.000
FGFR2-c	FGFR2-end	0.855	0.002
FOXO1-c	FOXO1-end	0.995	0.000
**Strong correlation**			
FGFR1-e	FOXO1-e	0.640	0.046
FGFR1-e	FOXO1-c	0.643	0.045
FGFR1-e	FOXO1-end	0.699	0.024
FGFR2-e	FGFR2-c	0.652	0.041
FOXO1-e	FGFR1-c	0.651	0.042
FOXO1-e	FGFR1-end	0.651	0.042
FGFR1-c	FOXO1-c	0.649	0.042
FGFR1-c	FOXO1-end	0.711	0.021
FOXO1-c	FGFR1-end	0.649	0.042
FGFR1-end	FOXO1-end	0.711	0.021
**Moderate correlation**			
FGFR2-e	FGFR2-end	0.472	0.169
**Weak correlation**			
FGFR1-e	FGFR2-c	-0.323	0.363
FGFR1-e	FGFR2-end	-0.248	0.489
FGFR2-e	FOXO1-e	0.306	0.391
FOXO1-e	FGFR2-c	0.288	0.420
FGFR1-c	FGFR2-c	-0.321	0.366
FGFR1-c	FGFR2-end	-0.247	0.492
FGFR2-c	FGFR1-end	-0.321	0.366
FOXO1-c	FGFR2-end	0.255	0.476
FGFR1-end	FGFR2-end	-0.247	0.492
FGFR2-end	FOXO1-end	0.208	0.563
**Very weak correlation**			
FGFR1-e	FGFR2-e	0.000	1.000
FGFR2-e	FGFR1-c	0.000	1.000
FGFR2-e	FOXO1-c	0.141	0.698
FGFR2-e	FGFR1-end	0.000	1.000
FGFR2-e	FOXO1-end	0.130	0.721
FOXO1-e	FGFR2-end	0.137	0.706
FGFR2-c	FOXO1-c	0.194	0.592
FGFR2-c	FOXO1-end	0.144	0.692

Abbreviations: FGFR1- fibroblast growth factor receptor 1, FGFR2- fibroblast growth factor receptor 2, FOXO1- forkhead box O.
Note: -e indicates "in the epithelium", -c indicates "in the connective tissue", -end indicates "in the endothelium".

Table S2. Correlations between factors in patients' palatine tissue samples based on Spearman's correlations analyses.

Factor 1	Factor 2	R	*p*-Value
**Very strong correlation**			
FGFR1-e	FGFR1-c	0.837	0.002
FGFR2-e	FGFR2-c	0.811	0.004
FOXO1-e	FOXO1-end	0.861	0.001
FGFR1-c	FGFR1-end	0.855	0.002
FGFR2-c	FGFR2-end	0.802	0.005
**Strong correlation**			
FGFR1-e	FGFR1-end	0.725	0.018
FGFR2-e	FGFR2-end	0.707	0.022
FOXO1-e	FGFR1-end	0.679	0.031
FOXO1-c	FOXO1-end	0.667	0.035
**Moderate correlation**			
FGFR2-e	FOXO1-end	-0.411	0.238
FOXO1-e	FGFR1-c	0.551	0.099
FOXO1-e	FOXO1-c	0.574	0.083
**Weak correlation**			
FGFR1-e	FGFR2-e	0.289	0.417
FGFR1-e	FOXO1-e	0.322	0.365
FGFR1-e	FOXO1-c	-0.360	0.306
FGFR1-e	FGFR2-end	0.244	0.496
FGFR2-e	FOXO1-e	-0.200	0.579
FGFR1-c	FOXO1-c	-0.215	0.551
FGFR1-c	FOXO1-end	0.269	0.452
FOXO1-c	FGFR2-end	-0.215	0.551
FGFR1-end	FGFR2-end	0.208	0.563
FGFR1-end	FOXO1-end	0.311	0.382
FGFR2-end	FOXO1-end	-0.323	0.363
**Very weak correlation**			
FGFR1-e	FGFR2-c	0.183	0.612
FGFR1-e	FOXO1-end	0.045	0.902
FGFR2-e	FGFR1-c	-0.012	0.974
FGFR2-e	FOXO1-c	-0.122	0.738
FGFR2-e	FGFR1-end	0.014	0.970
FOXO1-e	FGFR2-c	0.138	0.704
FOXO1-e	FGFR2-end	-0.083	0.819
FGFR1-c	FGFR2-c	-0.073	0.841
FGFR1-c	FGFR2-end	0.042	0.909
FGFR2-c	FOXO1-c	0.188	0.602
FGFR2-c	FGFR1-end	0.150	0.680
FGFR2-c	FOXO1-end	-0.094	0.796
FOXO1-c	FGFR1-end	-0.166	0.647

Abbreviations: FGFR1- fibroblast growth factor receptor 1, FGFR2- fibroblast growth factor receptor 2, FOXO1- forkhead box O.
Note: -e indicates "in the epithelium", -c indicates "in the connective tissue", -end indicates "in the endothelium".
